# Relationship between renal function and circulating microparticles, soluble P-selectin and E-selectin levels in atrial fibrillation

**DOI:** 10.1007/s11239-016-1427-3

**Published:** 2016-09-26

**Authors:** Yee Cheng Lau, Qinmei Xiong, Andrew D. Blann, Gregory Y. H. Lip

**Affiliations:** 1 University of Birmingham Institute of Cardiovascular Science, City Hospital, Dudley Road, Birmingham, UK; 2 Cardiovascular Department, The Second Affiliated Hospital of Nanchang University, Nanchang, China

**Keywords:** Atrial fibrillation, Chronic renal disease, Microparticles, Soluble P selectin, Soluble E selectin

## Abstract

Atrial fibrillation (AF) and chronic kidney disease are closely related, and any associated risk of stroke and thromboembolism due to AF is increased by concurrent renal dysfunction. The mechanism(s) for this include abnormalities in platelets and endothelial cells. We hypothesized relationships between levels of circulating platelet microparticles (PMPs, defined by CD42b), soluble P selectin (both reflecting platelet activation), soluble E-selectin (reflecting endothelial activation) and endothelial/platelet microparticles (EPMPs, defined by CD31) with progressive renal dysfunction. Blood samples were obtained from 160 anticoagulated AF patients. Microparticles were measured by flow cytometry, soluble E and P selectin levels by ELISA. Renal function was determined by estimated glomerular filtration rate (eGFR). EPMP levels demonstrated a linear increased trend across quartiles of eGFR (p = 0.034) and CKD stage (p < 0.001), and correlated with eGFR and serum creatinine (p < 0.01). PMPs, P-selectin and E-selectin levels were not significantly different across groupings of renal dysfunction, and no significant correlations with eGFR were evident (p = 0.186, p = 0.561, p = 0.746 respectively). Stepwise multivariable regression analysis demonstrated that worsening renal function was an independent predictor of EPMP levels (p < 0.001). In well-anticoagulated AF patients, there is potential relationship between endothelial function (as judged by elevated EPMP levels, with no change in PMPs) and renal function. Other markers of prothombotic state or cellular activation (PMP, P-selectin and E-selectin levels) were not significantly different across the various degree of renal dysfunction. Renal function must be addressed when measuring EPMP levels.

## Introduction

Non-valvular atrial fibrillation (AF) is associated with an elevated risk of ischaemic stroke and systemic thromboembolism [1, 2]. This risk is further increased by concurrent diagnosis of chronic kidney disease (CKD) or end-stage renal disease (ESRD) and results in a worse clinical outcome in terms of cardiovascular disease [3–5]. The precise mechanism(s) underlying the worse outcomes in AF patients with CKD and ESRD are incompletely understood and so require further investigation. One such aspect is circulating microparticles, known to be increased in dialysis dependent ESRD [6]. These microparticles are heterogenous vesicles, derived from cellular membrane where the parent cells have undergone necrosis, injury, apoptosis or activation [7, 8]. Depending on the nature of their parent cells (such as leukocytes, platelets and endothelial cells), different microparticles subsets possess a unique composition and content, which vary in their thrombotic potentials [9–11]. Thus, different microparticles subsets can not only mark pathology of their parent cells, but may also influence coagulation by directly facilitating formation of coagulation complexes or via modulation of tissue factor dependent pathways [12, 13].

Although microparticles levels are increased in ESRD and are linked to increased cardiovascular mortality [6, 14], a contradictory relationship exists between AF and levels of circulating microparticles [15, 16]. A potential relationship between worsening degrees of non-dialysis dependent renal dysfunction and microparticles amongst AF patients, as well as the subsequent effect on levels of various microparticle subsets, has yet to be investigated.

In this study, we hypothesised that, in patients at risk of a thrombotic events (that is, with AF) levels of platelet microparticles and/or endothelial/platelet microparticles would be associated inversely with renal function, as defined by the estimated glomerular filtration rate. To provide perspective for the microparticle aspect, we also measured levels of two plasma markers of platelet and endothelial biology, these being soluble P selectin and soluble E selectin respectively, both known to be abnormal in cardiovascular disease [17, 18].

## Patients and methods

### Subjects

All 160 subjects with non-valvular AF were recruited from routine out-patient clinics, and all had been taking oral anticoagulation (warfarin) for at least 12 weeks. Dose-adjustment for warfarin was done in specialised scientist-led anticoagulation clinics, achieving a stable international normalised ratio (INR) between two and three. INR was again determined on the day of testing to assess effective anticoagulation.

Exclusion criteria were age <18 years, diagnosis of valvular AF (severe rheumatic stenosis, metallic prosthetic valve, mitral/tricuspid ring repair), active or recent (<12 months) malignancy, active immunological disease, chronic liver disease, recent or chronic infections, chronic inflammatory disease, connective tissue disease, recent stroke/acute coronary syndrome (within 2 months), active bleeding, recent arterial/venous thrombosis or recent surgery, known haemophilia or thrombophilia (such as Factor V Leiden, Protein C/S/anti-thrombin deficiency, anti-phospholipid syndrome), use of an anti-platelet agent other than aspirin, and use of an oral anticoagulant other than warfarin. Standard clinical and demographic data were obtained.

A routine blood test was taken for to assess for renal function (urea, creatinine and electrolytes), with subsequent calculation of estimated glomerular filtration rate (eGFR) [19]. To aid clarity, patients with CKD stages four and five were merged into a single group [20]. In accordance with the Declaration of Helsinki, the project was approved by the Local Research Ethics Committee and written informed consent was obtained from each subject. Data were anonymised prior to statistical analysis.

### Laboratory methods

Blood samples were collected from a large antecubital vein using a 21-gauge needle directly into Vacutainer® tubes (Becton Dickinson, UK) containing 0.5 ml 3.2 % sodium citrate. For microparticle detection, platelet-poor plasma (PPP) was obtained after 15 min centrifugation of citrated blood at 2800*g* and further centrifugation of PPP at 13,000*g* for 2 min to remove residual cellular fragments to obtain platelet-free plasma (PFP) as per ISTH guidelines [20]. Aliquots of the plasmas were frozen at −70 °C for subsequent batch analysis and so had undergone a single-freeze thaw cycle.

PFP was initially incubated separately for 30 min with 0.5 μg of biotinylated anti-human CD42b antibody (Abcam, Cambridge, UK) for platelet-derived microparticles (PMP), or 0.5 μg of biotinylated anti-human antibody to CD31 (PECAM, present on both platelets and endothelial cells) (Abcam, Cambridge, UK) for mixed endothelial-derived microparticles (EPMPs). This was followed by a second incubation with 0.25 μg of Streptavidin-Alexa Fluor-647 nm-R-Phycoerythrin conjugate (Life Technology, Paisley, UK) for 30 min and then diluted with 990 μl filtered PBS (final dilution 1:100). MP analysis was promptly performed using the Apogee A50 flow cytometer (Apogee Flow Systems, High Wycombe, UK). Polystyrene beads of 110, 200, 500 nm and 1 μm diameter (Apogee Flow Systems) were used to set up the MP-size gate and small-size MP defined as events with size between 110 and 500 nm. Detailed instruction regarding gating selection has previously been described [21]. For enzyme-linked immunosorbent assay (ELISA) blood samples were centrifuged within 30 min from collection at 1500*g* for 20 min at 4 °C. The resultant plasma was then collected and stored at −70 °C until later batch processing by ELISA to measure soluble E-selectin and soluble P-selectin (R&D Systems, Abingdon, UK).

### Statistical analysis

Continuously variable data are expressed as mean and standard deviation (SD) or median and interquartile range (IQR) dependent on distribution. To demonstrate a step change in research indices, data were grouped by quartile of eGFR and also by the clinical tool of CKD stage, that being Stage 1 (eGFR ≥90 ml/min/1.73 m^2^), Stage 2 (eGFR 60–89), Stage 3 (eGFR 30–59) and Stage 4/5 combined (eGFR ≤29) [22]. When the groups are ordered it is not reasonable to compared each pair of groups with each other (as in an analysis of variance of groups that are independent), but instead consideration should be given whether there is a linear trend across the four groups. This was sought according to methods described by Altman [23]. However, as the eGFR has a natural continuous variation, correlations were also sought using Spearman’s method. Categorical indices were analysed by the Chi squared test. Stepwise regression analyses were performed to determine independent influences on research indices. All analyses were performed on Minitab 17, and p < 0.05 was considered as significant.

## Results

Clinical and demographic details of the 160 AF patients sorted by quartile of eGFR are shown in Table 1. There were no significant differences in INR, gender, most comorbidities, race (black vs. non-black), systolic blood pressure, nicotine use or concurrent antiplatelet use. As expected, there was worsening renal function with increasing CKD stage, age and creatinine level, but diastolic blood pressure and BMI were lowest in those with worse renal function. Diabetes was linked to worsening renal function.

**Table 1:** Clinical and demographic data

	eGFR				P for linear trend
	Quartile 1: best renal function	Quartile 2	Quartile 3	Quartile 4: worst renal function	
eGFR (ml/min/1.73)	87 (5)	70 (3)	56 (6)	30 (7)	<0.001
Age (years)	68 (12)	74.5 (8)	74 (7)	80 (8)	<0.001
Sex (m/f)	24/16	21/19	22/18	21/19	0.895
Race (non-black/black)	37/3	39/1	33/7	35/5	0.135
Weight (kg)	92 (24)	87 (22)	83.5 (15)	76 (21)	<0.001
Creatinine (µmol/l)	70 (13)	85 (14)	109 (25)	166 (60)	<0.001
INR	2.6 (0.8)	2.6 (0.7)	2.5 (0.8)	2.4 (0.6)	0.086
SBP (mmHg)	132 (18)	135 (17)	126 (24)	129 (18)	0.096
DBP (mmHg)	76 (13)	75 (12)	72 (13)	69 (9)	0.002
BMI (kg/m^2^)	30.3 (6.3)	31.0 (5.6)	29.5 (4.6)	28.1 (6.8)	0.027
Co-morbidities (yes/no)					
Ischaemic heart disease	12/28	15/25	16/24	23/17	0.082
Type 2 diabetes mellitus	14/26	11/29	17/23	24/16	0.022
Hypertension	33/7	34/6	33/7	35/5	0.914
Heart failure	12/28	12/28	15/25	21/19	0.124
COPD	9/31	4/36	5/35	9/31	0.296
Concurrent antiplatelet	2/38	2/38	4/36	4/36	0.696
Current smoker	2/38	1/39	1/39	2/38	0.875
CKD stage 1/2/3/4 (n)	23/17/0/0	0/40/0/0	0/12/28/0	0/0/17/23	<0.001
Median (IQR) CKD stage	1 (1–2)	2 (2–2)	3 (2–3)	4 (3–4)	<0.001

Data mean (standard deviation), number of subjects, or median and inter-quartile range (CKD stage)

*eGFR* estimated glomerular filtration rate, *INR* international normalised ratio, *SBP* systolic blood pressure, *DBP* diastolic blood pressure, *BMI* body mass index

EPMP levels increased sequentially across the quartiles of worsening renal function as defined by eGFR, but there were no differences in PMP, soluble P-selectin, or E-selectin levels (Table 2). The trend in EPMP across CKD stages 1–4/5 of 8.62 (3.94–18.23), 6.12 (1.62–12.78), 9.21 (1.82–23.17) and 21.05 (3.1-46.47) × 10^3^/µl respectively, although not completely linear, was nonetheless highly significant (p < 0.001).

**Table 2:** Microparticles, soluble P-selectin and soluble E-selectin levels according to quartiles of eGFR

	eGFR				P for linear trend
	Quartile 1	Quartile 2	Quartile 3	Quartile 4	
PMPs events/µl	281 (134–1348)	2123 (324–4079)	1476 (82–5041)	209 (29–2043)	0.897
EPMPs events/10^3^/µl	5.18 (0.74–12.35)	6.52 (4.22–11.67)	6.09 (0.41–24.45)	21.05 (6.43–29.71)	0.034
Soluble P selectin (ng/ml)	9.1 (7.3–11.0)	9.0 (7.6–10.4)	9.1 (7.2–11.4)	8.3 (6.2–10.8)	0.368
Soluble E selectin (ng/ml)	39 (29–105)	39 (27–74)	43 (29–63)	39 (26–45)	0.930

Data presented as median and IQR

*PMPs* platelet microparticles, *EPMPs* endothelial/platelet microparticles

There was a significant negative correlation between eGFR and EPMP, and a positive correlation between serum creatinine and EPMP (r = 0.209, p = 0.008) (Table 3). There were no significant correlations between eGFR and PMP, soluble P-selectin or soluble E-selectin levels. Using stepwise regression analysis of indices in Table 1 with p < 0.05, the only independent predictor EPMP was the eGFR (p < 0.001). Other demographic factors and comorbidities did not have any significant impact on EPMP levels.

**Table 3:** Spearman correlations of the eGFR with microparticles, P-selectin and E-selectin levels

	r_s_	p
Platelet microparticles	0.095	0.234
Endothelial/platelet microparticles	−0.319	<0.001
Soluble P selectin	0.086	0.279
Soluble E selectin	0.133	0.094

*r*_*s*_ Spearman’s correlation coefficient

## Discussion

Renal function is an emerging additional risk factor for cardiovascular disease [24]. As AF and CKD are closely linked, there is a strong need to identify potential markers that enable assessment of the decline of renal function and alteration(s) of thrombotic potential [1–5]. We demonstrate that among AF patients receiving oral anticoagulation, progressive worsening of renal function is associated with a linear trend of increasing levels of EPMP. Reduced diastolic blood pressure in patients with greatest renal dysfunction may be due to the higher proportion of patient with ischaemic heart disease and the likelihood that they will be more intensively treated. Similarly, the increasing frequency of diabetes across the groups is very likely to be due to diabetic nephropathy. Nevertheless, neither of these factors were selected on multivariate analysis and renal function emerged as the only independent determinant of EPMP levels.

Our current finding confirms previous studies finding increased microparticles in renal dysfunction/failure [6, 25, 26]. However, our data contrasts with the lack of positive findings in other studies, possible because of the use of different antibodies, such as to CD144, CD146 and CD41 [26], annexin-V [27] and/or to CD69E [28], and so defining different species of MP [8], in demographic and co-morbidities (the approximate mean age of the patients of Fauvre et al. [26] is 59 years, of Lu et al. [28] is 47 years, of Chen et al. [27] it is 70 years, whilst in our patients it is 74 years), racial background (90 % of our patients were of European descent whilst we presume the majority of those of Chen et al. and Lu et al. were from the Far East), and differences in ELISA reagents.

As EPMPs may arise from endothelial cells and platelets in response to damage/activation, increased levels may reflect pathology of either or both parent cells. We used CD31, also known as PECAM, is found on the surface of both platelets and endothelial cells [29–32]. However, the failure of ‘pure’ PMPs to associate with any renal function index provide support for the hypothesis that EPMPs are more likely to reflect endothelial pathology [33]. The presence of greater vascular and endothelial injury associated with (or as a result of) progressive renal dysfunction will cause alteration to EMP levels. Hence, this study also extends previous work by demonstrating a progressive, step-wise, increase in EPMP levels among those with worsening degrees of renal failure. Nevertheless, elevated CD31/annexin-V EPMP levels are present in acute coronary syndromes, may be a surrogate of cellular injury due to cardiovascular risk diseases, and correlate with cardiovascular outcomes [34–36], but this can be accounted for in our study by the similar comorbidities.

Our study demonstrates the lack of significant change in PMP levels in relation with worsening renal function among AF patients. Previous studies [15, 37] have shown that PMP levels are affected less by arrhythmia and more to underlying cardiovascular diseases. Lu et al. [28] also found that CD41-defined PMPs and soluble P selectin were progressively lower in those with the worse CKD, in contrast to our data which found no such relationship. The lack of alterations in soluble P-selectin and E-selectin levels across subjects with worsening renal function suggests that these biomarkers of platelet and endothelial cell activation may be less affected by renal (dys)function.

Limitations of our study include lack of data regarding the potential roles of other microparticles besides PMP and EPMP, such as ‘pure’ endothelial (e.g. as defined by CD62E, CD144, CD146, etc. [8, 26, 38]), or leukocyte subsets. Furthermore, all patients were taking warfarin, so that extrapolation of non-AF and/or non-anticoagulated groups may be difficult, and without a platelet count we cannot relate this index to PMPs or soluble P selectin. However, we have previously been unable to link soluble P selectin with platelet count [39, 40]. Further studies should be performed to investigate their potential involvement in the relationship of progressive renal dysfunction and AF.

In conclusion, among well-anticoagulated AF patients, there is relationship between endothelial function (as judged by EPMPs) and renal function. Other markers of prothombotic state or cellular activation, such as PMP, P-selectin and E-selectin levels were not significantly different across the various degree of renal dysfunction.

## References

[1] Wolf PA, Dawber TR, Thomas HE, Kannel WB (1978). Epidemiologic assessment of chronic atrial fibrillation and risk of stroke: the Framingham study. Neurology.

[2] Yamashita Y, Hamatani Y, Esato M, Chun YH, Tsuji H, Wada H (2016). Clinical characteristics and outcomes in extreme elderly (age ≥85) Japanese patients with atrial fibrillation: the Fushimi AF registry. Chest.

[3] Go AS, Fang MC, Udaltsova N, Chang Y, Pomernacki NK, Borowsky L (2009). Impact of proteinuria and glomerular filtration rate on risk of thromboembolism in atrial fibrillation: the anticoagulation and risk factors in atrial fibrillation (ATRIA) study. Circulation.

[4] Olesen JB, Lip GY, Kamper AL, Hommel K, Kober L, Lane DA (2012). Stroke and bleeding in atrial fibrillation with chronic kidney disease. New Engl J Med.

[5] Bonde AN, Lip GY, Kamper AL, Hansen PR, Lamberts M, Hommel K (2014). Net clinical benefit of antithrombotic therapy in patients with atrial fibrillation and chronic kidney disease: a nationwide observational cohort study. J Am Coll Cardiol.

[6] Amabile N, Guerin AP, Leroyer A, Mallat Z, Nguyen C, Boddaert J (2005). Circulating endothelial microparticles are associated with vascular dysfunction in patients with end-stage renal failure. J Am Soc Nephrol.

[7] Shantsila E, Montoro-Garcia S, Gallego P, Lip GY (2014). Circulating microparticles: challenges and perspectives of flow cytometric assessment. Thromb Haemostasis.

[8] Nomura S (2016). Microparticle and atherothrombotic diseases. J Atheroscler Thromb.

[9] Sinauridze EI, Kireev DA, Popenko NY, Pichugin AV, Panteleev MA, Krymskaya OV (2007). Platelet microparticle membranes have 50- to 100-fold higher specific procoagulant activity than activated platelets. Thromb Hemostasis.

[10] Jy W, Jimenez JJ, Mauro LM, Horstman LL, Cheng P, Ahn ER (2005). Endothelial microparticles induce formation of platelet aggregates via a von Willebrand factor/ristocetin dependent pathway, rendering them resistant to dissociation. J Thromb Haemostasis.

[11] Falati S, Liu Q, Gross P, Merrill-Skoloff G, Chou J, Vandendries E (2003). Accumulation of tissue factor into developing thrombi in vivo is dependent upon microparticle P-selectin glycoprotein ligand 1 and platelet P-selectin. J Exp Med.

[12] Del Conde I, Shrimpton CN, Thiagarajan P, Lopez JA (2005). Tissue-factor-bearing microvesicles arise from lipid rafts and fuse with activated platelets to initiate coagulation. Blood.

[13] Steppich B, Mattisek C, Sobczyk D, Kastrati A, Schomig A, Ott I (2005). Tissue factor pathway inhibitor on circulating microparticles in acute myocardial infarction. Thromb Haemostasis.

[14] Amabile N, Guerin AP, Tedgui A, Boulanger CM, London GM (2012). Predictive value of circulating endothelial microparticles for cardiovascular mortality in end-stage renal failure: a pilot study. Nephrol Dialysis Transplant.

[15] Choudhury A, Chung I, Blann AD, Lip GY (2007). Elevated platelet microparticle levels in nonvalvular atrial fibrillation: relationship to P-selectin and antithrombotic therapy. Chest.

[16] Jesel L, Abbas M, Toti F, Cohen A, Arentz T, Morel O (2013). Microparticles in atrial fibrillation: a link between cell activation or apoptosis, tissue remodelling and thrombogenicity. Int J Cardiol.

[17] Blann AD, Nadar SK, Lip GY (2003). The adhesion molecule P-selectin and cardiovascular disease. Eur Heart J.

[18] Roldán V, Marín F, Lip GY, Blann AD (2003). Soluble E-selectin in cardiovascular disease and its risk factors. A review of the literature. Thromb Haemost.

[19] Levey AS, Bosch JP, Lewis JB, Greene T, Rogers N, Roth D (1999). A more accurate method to estimate glomerular filtration rate from serum creatinine: a new prediction equation. Modification of diet in renal disease study group. Ann Intern Med.

[20] Lacroix R, Judicone C, Poncelet P, Robert S, Arnaud L, Sampol J (2012). Impact of pre-analytical parameters on the measurement of circulating microparticles: towards standardization of protocol. J Thromb Haemost.

[21] Kidney Disease Improving global outcomes (KDIGO) CKD Work Group (2013) KDIGO 2012 clinical practice guideline for the evaluation and management of chronic kidney disease. Kidney Int (Suppl 3):1–150

[22] Montoro-Garcia S, Shantsila E, Orenes-Pinero E, Lozano ML, Lip GY (2012). An innovative flow cytometric approach for small-size platelet microparticles: influence of calcium. Thromb Haemostasis.

[23] Altman DG (1991). Practical statistics for medical research.

[24] Kundhal K, Lok CE (2005). Clinical epidemiology of cardiovascular disease in chronic kidney disease. Nephron Clin Pract.

[25] Almquist T, Mobarrez F, Jacobson SH, Wallen H, Hjemdahl P (2016). Effects of lipid-lowering treatment on circulating microparticles in patients with diabetes mellitus and chronic kidney disease. Nephrol Dial Transplant.

[26] Faure V, Dou L, Sabatier F, Cerini C, Sampol J, Berland Y (2006). Elevation of circulating endothelial microparticles in patients with chronic renal failure. J Thromb Haemost.

[27] Chen YL, Chen CH, Wallace CG, Wang HT, Yang CC, Yip HK (2015). Levels of circulating microparticles in patients with chronic cardiorenal disease. J Atherosclerosis Thrombosis.

[28] Lu GY, Xu RJ, Zhang SH, Qiao Q, Shen L, Li M (2015). Alteration of circulatory platelet microparticles and endothelial microparticles in patients with chronic kidney disease. Int J Clin Exp Med.

[29] Metzelaar MJ, Korteweg J, Sixma JJ, Nieuwenhuis HK (1991). Biochemical characterization of PECAM-1 (CD31 antigen) on human platelets. Thromb Haemost.

[30] Mei H, Campbell JM, Paddock CM, Lertkiatmongkol P, Mosesson MW, Albrecht R (2014). Regulation of endothelial cell barrier function by antibody-driven affinity modulation of platelet endothelial cell adhesion molecule-1 (PECAM-1). J Biol Chem.

[31] Privratsky JR, Newman PJ (2014). PECAM-1: regulator of endothelial junctional integrity. Cell Tissue Res.

[32] Kellermair J, Redwan B, Alias S, Jabkowski J, Panzenboeck A, Kellermair L (2013). Platelet endothelial cell adhesion molecule 1 deficiency misguides venous thrombus resolution. Blood.

[33] Wang JM, Huang YJ, Wang Y, Xu MG, Wang LC, Wang SM (2007). Increased circulating CD31+/CD42− microparticles are associated with impaired systemic artery elasticity in healthy subjects. Am J Hypertens.

[34] Bernal-Mizrachi L, Jy W, Jimenez JJ, Pastor J, Mauro LM, Horstman LL (2003). High levels of circulating endothelial microparticles in patients with acute coronary syndromes. Am Heart J.

[35] Werner N, Wassmann S, Ahlers P, Kosiol S, Nickenig G (2006). Circulating CD31+/annexin V+ apoptotic microparticles correlate with coronary endothelial function in patients with coronary artery disease. Arterioscl Thromb Vascular Biol.

[36] Sinning JM, Losch J, Walenta K, Böhm M, Nickenig G, Werner N (2011). Circulating CD31+/Annexin V+ microparticles correlate with cardiovascular outcomes. Eur Heart J.

[37] Ederhy S, Di Angelantonio E, Mallat Z, Hugel B, Janower S, Meuleman C (2007). Levels of circulating procoagulant microparticles in nonvalvular atrial fibrillation. Am J Cardiol.

[38] Dursun I, Yel S, Unsur E (2015). Dynamics of circulating microparticles in chronic kidney disease and transplantation: is it really reliable marker?. World J Transplant.

[39] Nadar SK, Blann AD, Kamath S, Beevers DG, Lip GY (2004). Platelet indexes in relation to target organ damage in high-risk hypertensive patients: a sub-study of the Anglo-Scandinavian Cardiac Outcomes Trial (ASCOT). J Am Coll Cardiol.

[40] Caine GJ, Blann AD (2003). Soluble P-selectin should be measured in citrated plasma, not in serum. Br J Haematol.

